# Outbreak of Vancomycin-Resistant Enterococcus in a NICU: Insights into Molecular Detection and Infection Control †

**DOI:** 10.3390/microorganisms13040822

**Published:** 2025-04-04

**Authors:** Francesco Peracchi, Giovanna Travi, Alice Proto, Elena Nicolini, Andrea Busni, Luca Mezzadri, Livia Tartaglione, Alessandra Bielli, Elisa Matarazzo, Giorgia Casalicchio, Cecilia Del Curto, Roberto Rossotti, Marco Merli, Chiara Vismara, Fulvio Crippa, Stefano Martinelli, Massimo Puoti

**Affiliations:** 1 School of Medicine and Surgery, University of Milano-Bicocca, Piazza Dell’Ateneo Nuovo 1, 20126 Milano, Italy; l.mezzadri3@campus.unimib.it (L.M.); massimo.puoti@unimib.it (M.P.); 2 Infectious Diseases Unit, ASST Grande Ospedale Metropolitano Niguarda, Piazza Dell’Ospedale Maggiore 3, 20162 Milan, Italy; roberto.rossotti@ospedaleniguarda.it (R.R.); marco.merli@ospedaleniguarda.it (M.M.); fulvio.crippa@ospedaleniguarda.it (F.C.); 3 Neonatology and Neonatal Intensive Care Unit, ASST Grande Ospedale Metropolitano Niguarda, Piazza Dell’Ospedale Maggiore 3, 20162 Milan, Italy; alice.proto@ospedaleniguarda.it (A.P.); stefano.martinelli@ospedaleniguarda.it (S.M.); 4 Microbiology and Virology, Facoltà di Medicina e Chirurgia, Università degli Studi di Milano, Via Festa del Perdono 7, 20122 Milan, Italy; elena.nicolini@ospedaleniguarda.it; 5 Clinical Microbiology, ASST Grande Ospedale Metropolitano Niguarda, Piazza Dell’Ospedale Maggiore 3, 20162 Milan, Italy; andrea.busni@ospedaleniguarda.it (A.B.); livia.tartaglione@ospedaleniguarda.it (L.T.); alessadra.bielli@ospedaleniguarda.it (A.B.); elisa.matarazzo@ospedaleniguarda.it (E.M.); giorgia.casalicchio@ospedaleniguarda.it (G.C.); chiara.vismara@ospedaleniguarda.it (C.V.); 6 ASST Grande Ospedale Metropolitano Niguarda, Piazza Dell’Ospedale Maggiore 3, 20162 Milan, Italy; cecilia.delcurto@ospedaleniguarda.it

**Keywords:** VRE, colonization, molecular test, pathogenicity, infection control

## Abstract

Vancomycin-resistant enterococci (VRE) are a major cause of healthcare-associated infections (HAIs). However, the clinical significance of VRE colonization and the subsequent risk of VRE infection in hospitalized patients are not fully established. Prolonged hospital stays have been observed in neonates colonized by VRE. The mortality rate in pediatric patients with VRE infections ranges from 0% to 42% in both endemic and outbreak settings, often occurring in VRE-colonized neonates. Host and bacterial factors associated with a worse outcome are not fully understood yet. We describe an outbreak of VRE colonization in 21 newborns admitted to our neonatal intensive care unit in January 2024. Microbiological analyses on rectal swabs were performed using molecular testing and culture. Results: In January, VRE was first detected in the urine culture of a 3-week-old patient, followed by a subsequent positive rectal swab result. In accordance with our infection control policy, all the NICU patients were tested, leading to the identification of another 12 colonized patients. The implementation of molecular testing led to rapid VRE identification and the subsequent isolation of colonized neonates, which promptly contained the outbreak. The median time from NICU admission to colonization was 34 (6–37) days. Only one patient developed a CVC-related bloodstream infection, which was successfully treated with linezolid and CVC removal. No VRE-related deaths occurred, even among three patients who underwent abdominal surgery (one gastroschisis, one incarcerated abdominal hernia, and one umbilical hernia) and one patient with necrotizing enterocolitis. Our data show a low infection rate (4%) among VRE-colonized patients (4%) during a NICU outbreak. The rapid identification of multidrug-resistant genes by molecular testing may be implemented in specific settings to enable timely patient identification, adopt infection control measures, and administer appropriate antimicrobial therapy.

## 1. Introduction

*Enterococcus* spp. are commensal bacteria of the gastrointestinal tract, colonizing the gut within hours of birth [1]. The two most common species associated with infections in humans are *E. faecalis* and *E. faecium*. While healthcare-associated infections have historically been caused by *E. faecalis*, the prevalence of *E. faecium* is now increasing, driven by the expansion of clonal complex **17** [2].

A major challenge in treating enterococcal infections is their increasing resistance to antibiotics such as aminopenicillins, glycopeptides, aminoglycosides, and oxazolidinones, further complicated by the intrinsic resistance of *Enterococcus* spp. to many antibiotic classes [2,3]. The hospital-associated *E. faecium* clade with *pbp5-*R, the allele encoding the ampicillin-resistant version of PBP5 (penicillin-binding protein 5), is the cause of a vast majority of hospital-acquired enterococcal outbreaks [4]. Vancomycin resistance in *E. faecium* is mediated by a set of regulatory, functional, and accessory genes forming the *van operon* [5]. Currently, the most clinically significant resistant genes are *van A, van B*, and *van M*, which confer intermediate to high levels of resistance and are located on mobile genetic elements, allowing horizontal gene transfer to other bacteria [6].

*Enterococcus* may cause various infections, including bloodstream infections (BSIs), urinary tract infections, abdominal infections, surgical site infections, endocarditis, meningitis, and device-related infections [2,4,7].

*E. faecium*, particularly vancomycin-resistant *Enterococcus* (VRE), is usually involved in healthcare associated infections. These infections are frequently preceded by asymptomatic colonization, where bacteria reside in the gastrointestinal tract without causing clinical symptoms [8,9]. Studies have shown that a significant proportion of patients in healthcare settings are colonized, putting them at an elevated risk for subsequent infections, which leads to increased morbidity and mortality. Although VRE may be less virulent than some other multidrug-resistant organisms, limited treatment options remain a serious concern, particularly for vulnerable patients, for whom infections are a major cause of mortality [10].

In children, enterococcal infections are frequently healthcare-associated, typically occurring after chemotherapy or antimicrobial treatment [2]. In neonatal intensive care units (NICUs), reported VRE colonization rates vary significantly from 1.9% to as high as 42.2%, depending on the geographic region and local epidemiological conditions [11,12].

Despite these findings, the clinical significance of VRE colonization and its subsequent risk of infection in hospitalized patients are not fully elucidated. Neonates represent the most affected group during VRE outbreaks [7,13,14]; however, the host and bacterial factors that contribute to infection development and adverse outcomes remain incompletely understood. Prolonged hospitalization has been identified as a key risk factor for the progression from VRE colonization to infection, particularly in neonates with hospital stays exceeding 30 days [15]. Among neonates colonized by VRE, mortality rates have been reported to range from 0% to 42% [7,13], primarily in endemic and outbreak settings. These findings underscore the importance of understanding the dynamics of VRE colonization and implementing effective infection control measures to mitigate risks in this vulnerable population.

Moreover, VRE transmission is believed to occur through contact with healthcare workers’ hands or the hospital environment [16]. Neonates requiring intensive care are particularly vulnerable to colonization, and VRE can spread rapidly within this patient group.

Based on these observations, the rapid identification of colonized patients and infection control measures for reducing colonization is crucial.

Here, we describe an outbreak of VRE colonization in 21 newborns admitted to our neonatal intensive care unit, focusing on infection control measures adopted to contain the outbreak and underscoring the low pathogenicity of VRE, even within a high-risk population.

## 2. Materials and Methods

### 2.1. Hospital Setting

This study was conducted in the neonatal intensive care unit of a tertiary referral university hospital. The NICU consists of two large open-space rooms (75 and 78 m^2^) with capacities of 8 and 10 beds, respectively, and two isolation rooms (15 and 16 m^2^). Each isolation room is specifically equipped to care for neonates requiring strict infection control measures. The unit covers a total area of approximately 184 m^2^, providing an average of 7.65 m^2^ per bed in the open-space room and 15.5 m^2^ per bed in the isolated area.

Each bed is outfitted with advanced neonatal care equipment, including incubators with thermal regulation, multiparametric monitoring devices, mechanical ventilators, and intravenous therapy systems. Additional features include phototherapy units for the management of neonatal jaundice and bedside access to portable imaging equipment (i.e., ultrasound and X-ray).

Access to the unit is restricted to parents, who are allowed entry 24 h a day, following strict hygiene protocols. Healthcare personnel strictly adhere to infection control measures, including the use of personal protective equipment (PPE) and adherence to hand hygiene protocols, to minimize the risk of cross-contamination.

The staffing ratio is typically one nurse for every 2–3 neonates. However, a 1:1 nurse-to-patient ratio is implemented in cases of severe clinical instability or when intensive interventions are required. A multidisciplinary team, including neonatologists, specialized nurses, cardiologists, surgeons, etc., collaborates to provide comprehensive care. Regular case discussions and updates ensure optimal decision-making tailored to each patient’s needs.

Environmental factors, such as controlled air circulation and High-Efficiency Particulate Air (HEPA) filtration systems, are in place to maintain a sterile environment and limit airborne transmission of pathogens. Surveillance cultures performed every 14 days, and periodic environmental monitoring are part of the infection prevention strategy, particularly critical during outbreaks of multidrug-resistant organisms.

### 2.2. Study Design, Patients and Data Collection

A single-center retrospective study was performed, including neonatal patients with VRE colonization admitted to the NICU at Niguarda Hospital in Milan from January 2024 to April 2024.

The medical records of the patients identified as colonized or infected with VRE were reviewed for detailed clinical characteristics. Demographic, clinical, and biochemical data were collected from the hospital’s electronic records.

### 2.3. Definitions

VRE colonization was defined as a positive rectal swab obtained during routine surveillance in a patient in the absence of any clinical specimens yielding VRE. Systemic VRE infection was defined as the isolation of VRE from a sterile specimen, accompanied by signs and symptoms of infection in a patient previously colonized with VRE.

### 2.4. Surveillance Program

Patients admitted to our NICU are routinely screened with a rectal swab for the presence of extended-spectrum beta-lactamase (ESBL) producing *Enterobacterales* and carbapenem-resistant organisms within 48–72 h of admission and weekly thereafter.

The detection of vancomycin-resistant *Enterococcus* (VRE) in a urine culture, conducted based on clinical indications for a 3-week-old neonate and later confirmed by a positive rectal swab, led to the initiation of active VRE screening in the NICU. Following the identification of this index case, rectal swabs were immediately collected from all neonates currently admitted to the NICU to retrospectively identify additional colonized patients.

For new admissions during the outbreak period, molecular screening was implemented prospectively upon admission to ensure early detection. By the end of January 2024, in alignment with recommendations from the hospital’s Infection Control Committee, molecular screening for VRE using rectal swabs was formally established as part of routine surveillance for all new NICU admissions.

### 2.5. Microbiological Procedure

#### Culture and Susceptibility Test

Cultures (Fecal swab, Copan) were conducted on rectal swabs using chromogenic plates (chromID® VRE, Biomèrieux, Marcy-l’Etoile, France) (incubation in air, 35 ± 1 °C, 48 h). All suspected colonies were identified using MALDI-TOF (Bruker Daltonics, Billerica, MA, USA). Antimicrobial susceptibility tests were conducted using a PM-E37 microdilution panel on the MicroScan WalkAway 96 Plus semi-automated system (Beckman Coulter, Brea, CA, USA). Vancomycin- and teicoplanin-resistant phenotypes were verified with the gradient diffusion method (ETEST® Vancomycin, ETEST® Teicoplanin, Biomèrieux, Marcy-l’Etoile, France) on a Mueller Hinton II Agar plate (Liofilchem, Roseto degli Abruzzi, Italy) (incubation in air, 35 ± 1 °C).

All phenotypic results were interpreted according to the European Committee on Antimicrobial Susceptibility Testing (EUCAST) breakpoint. (The European Committee on Antimicrobial Susceptibility Testing. Breakpoint tables for interpretation of MICs and zone diameters. Version 14.0, 2024. http://www.eucast.org, accessed on 14 February 2025)

### 2.6. Molecular Test

Nucleic acid extraction and PCR setup were carried out using the NIMBUS and/or STARlet (Seegene, Seul, South Korea) automated system. Amplification was performed using CFX96 and C1000 thermal cyclers (BioRad, Hercules, CA, USA) and multiplex real-time PCR with an Allplex Entero-DR Assay (Seegene, Seul, South Korea) kit. This assay simultaneously detects vancomycin-resistant genes (*van A* and *van B*), carbapenemase genes (*blaVIM*, *blaNDM*, *blaKPC*, *blaOXA-48 like*, and *blaIMP*), and the *blaCTX-M* extended-spectrum beta-lactamase gene. Results were interpreted using the SEEGENE system.

### 2.7. Statistical Analysis

Descriptive statistics were used to summarize the features of the study population, using the median and interquartile range (IQR) for continuous variables, and absolute and relative [%] values for categorical variables.

## 3. Results

### 3.1. Demographic and Clinical Characteristics

Twenty-one consecutive patients with VRE colonization were included. All patients were admitted to the NICU. Their demographic and clinical characteristics are shown in Table 1. The median age at admission was 6 (1–1) days and the median body weight at birth was 1879 g (1150–2360 g). Sixteen (76%) patients were premature, with a gestational age of 33 + 1 day (31 + 3; 36 + 6). The most common reasons for NICU admission were prematurity (76%) and congenital heart disease (33%).

Seven patients (33%) underwent surgery before NICU admission, including three (14%) who had abdominal surgery. Eight (38%) patients received prior antimicrobial treatment due to BSIs, mainly related to *Staphylococcus* spp. infections.

### 3.2. Outbreak Management

In January 2024, vancomycin-resistant *Enterococcus* was first identified in the urine culture of a 3-week-old neonate, evaluated based on clinical indications, and subsequently confirmed by a positive rectal swab culture.

#### 3.2.1. Isolation and Surveillance

According to our infection control policy, the first colonized patient was placed under contact precautions, including the use of water-repellent gowns and gloves. Contact precautions were initially extended to non-colonized patients. A wide screening of all NICU patients, including those transferred to other units, identified 12 additional colonized patients. In order to restrict the cases and resolve the outbreak, the Clinical Microbiology Department together with the hospital’s Infection Control Committee decided to switch from conventional cultural-based testing to molecular testing. Moreover, to limit the spread of VRE, every new NICU admission was screened using molecular testing on rectal swabs for VRE. During the first 6–8 weeks of the outbreak, active surveillance was conducted three times per week using molecular testing. After the stabilization of cases, surveillance was reduced to twice per week, alternating between molecular and culture tests. All newly hospitalized patients underwent molecular testing and were placed under pre-emptive contact isolation pending results.

#### 3.2.2. Environmental Cleaning Enhancements

The NICU area with positive neonates was immediately sanitized, and colonized patients were physically separated from non-colonized patients. Separate cleaning carts were assigned to the sub-intensive and clean area of the NICU to prevent cross-contamination. Environmental swabs were taken to ensure adequate sanitization.

#### 3.2.3. Infection Control Measures and Reorganization of the Ward

To strengthen infection control and minimize the risk of additional colonization, the following measures were implemented:

1. Temporary closure of NICU admissions from 30 January to 12 February.
2. Physical unit separation of the NICU (Area A) from the sub-intensive care unit (Area B) using closed, fire-rated REI.
3. Staff cohorting to prevent cross-contact with nurses performing a key role.
4. Enhanced hand hygiene protocol, including training and direct observation of hand hygiene compliance from 31 January to 15 April.
5. Improved diaper management, including immediate handwashing after diaper changes and contact with patients, glove removal, and avoiding overflowing diaper bins.

The use of molecular testing for prompt VRE identification, with subsequent contact isolation of colonized neonates and rapid behavioral and organizational interventions, promptly resolved the outbreak.

The decision to reopen the NICU by 12 February was based on two key factors: some colonized newborns had already been discharged, and positive neonates still hospitalized had been assigned to one of two inpatient rooms. The second room was reserved for newborns who had tested negative on at least three consecutive molecular swabs and still required care. This arrangement allowed available beds in the non-isolated room to accommodate new admissions.

Seven patients were dismissed by March, and the outbreak was officially resolved in April.

In September 2024, two imported cases of VRE colonization were identified at admission through molecular testing screening, allowing for immediate isolation and prevention of a new outbreak.

### 3.3. Infections Course

Only one patient (5%) developed a central venous catheter (CVC)-associated BSI, which was successfully treated with a 7-day course of linezolid and CVC removal. He underwent rapid clinical improvement and microbiological resolution, with negative blood cultures after 48 h of antibacterial therapy. The time from colonization to infection was 6 days.

The time from NICU admission to the first detection of VRE colonization was 34 (6–37) days.

No other VRE-related infections or deaths were recorded, despite three patients undergoing abdominal surgery (one for gastroschisis, one for incarcerated abdominal hernia, and one for umbilical hernia), one patient developing necrotizing enterocolitis, and four patients undergoing cardio-thorax surgery.

Only one patient died, 30 days after VRE detection, from surgical complications; the death was not VRE-related.

**Figure 1:**
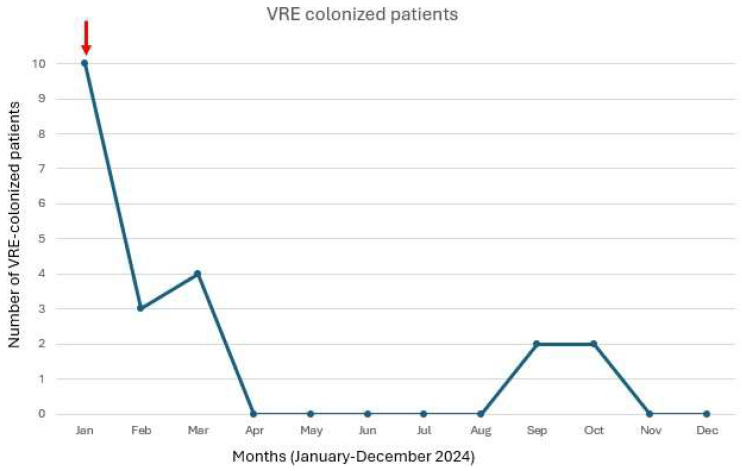
Number of VRE colonized patients over time in the neonatal intensive care unit. The figure shows the monthly number of neonates colonized with vancomycin-resistant *Enterococcus* in the neonatal intensive care unit during 2024. The red arrow indicates the introduction of polymerase chain reaction (PCR) testing for VRE detection in January 2024.

The outbreak course is shown in Figure 1.

### 3.4. Antimicrobial Susceptibility

As expected, all 21 tested strains exhibited resistance to both ampicillin and vancomycin. Furthermore, all strains were resistant to teicoplanin, indicating the likely presence of either the *van A* or *van M* resistance mechanism. All tested VRE strains were found to be susceptible to linezolid. Additionally, molecular testing of eight strains confirmed the presence of the *van A* gene. Microbiological characteristics and antibiograms are shown in Table 2.

## 4. Discussion

Vancomycin-resistant *Enterococcus* represents a significant challenge in healthcare, especially in high-risk settings, such as neonatal intensive care units, where neonates are particularly vulnerable.

Despite the high-risk environment, our cohort demonstrated a low incidence of severe infections and no VRE-related mortality, highlighting the effectiveness of early detection and rigorous infection control measures.

### 4.1. VRE Colonization and Pathogenicity

The pathogenicity of VRE is multifactorial, typically characterized by asymptomatic colonization, which serves as a reservoir for potential infection and transmission. The limited treatment options for VRE are particularly concerning for vulnerable populations, such as preterm neonates, for whom infections remain a leading cause of mortality. Although VRE virulence is relatively low compared to other multidrug-resistant organisms, the lack of effective therapies increases the risk for these high-risk patients [10]. Indeed, mortality rates associated with VRE infections are significantly higher than those related to vancomycin-sensitive *Enterococcus* infections [12].

VRE colonization prevalence varies widely across healthcare settings. In NICUs, colonization rates range from 1.9% to 39.9%, particularly during outbreaks [11]. A study conducted in northern Iran reported a colonization rate of 42.2% among neonates admitted to NICUs, which is significantly higher than the rates observed in other countries, such as the United States (3.6%) and Turkey (14.6%) [12].

Several factors contribute to VRE colonization and infection in NICUs. Prematurity, low birth weight, prolonged hospitalization, and prenatal antibiotic use are well-established risk factors [17,18,19,20]. The use of broad-spectrum antibiotics, particularly glycopeptides and cephalosporins, creates selective pressure that favors VRE colonization [16]. Additional contributing factors include the use of mechanical ventilation, the presence of central lines, and the administration of parenteral nutrition [19,21]. Interestingly, probiotics containing *Lactobacillus* species have been associated with an increased risk of VRE colonization, possibly due to the horizontal gene transfer of resistance genes during microbiota modulation [22]. Patiyan Andersson et al. [12] demonstrated that gestational age remains an independent significant risk factor for VRE colonization in multivariate analysis, even after adjusting for various other risk factors. In our cohort, 76% of neonates were born prematurely, and 38% had prior exposure to antimicrobial therapy, primarily for bloodstream infections. These findings corroborate available data showing that premature neonates and those with prior antimicrobial exposure are particularly susceptible to VRE colonization, reinforcing gestational age as a key risk factor in this vulnerable population [18].

In our study, only one of the 21 colonized neonates (5%) developed a clinical infection, which was a central venous catheter (CVC)-related bloodstream infection. This infection rate aligns with previous studies showing that 3–10% of VRE-colonized neonates in NICUs develop invasive infections [14,22]. Interestingly, a study by Marom et al. [15] found no infections among 49 colonized neonates during a similar outbreak.

Among VRE-colonized neonates, prolonged hospitalizations, particularly those exceeding 30 days, have been identified as a risk factor for the development of VRE infection [22]. Similarly, Sutcu et al. [13] identified long hospital stays (≥30 days) and glycopeptide use after VRE colonization as independent risk factors for systemic VRE infections.

The low rate of infection progression and the absence of VRE-related mortality in our cohort underscore the crucial impact of early and stringent infection control measures in preventing negative outcomes.

### 4.2. Molecular Testing

The widespread dissemination of multidrug-resistant organisms (MDROs) has sparked considerable debate regarding the utility of screening tests followed by the isolation of positive cases. Evidence has demonstrated that active surveillance testing to identify patients colonized with MDROs is associated with a significant reduction in MDRO acquisition during hospitalization, although results have been inconsistent [23]. Notably, some studies have highlighted how active surveillance cultures, combined with contact precautions for colonized patients, contributed to the decline of methicillin-resistant *Staphylococcus aureus* (MRSA) or VRE prevalence [24,25,26]. Early detection of MDRO-colonized patients is crucial for implementing timely interventions to prevent further spread [26] or resolve outbreaks, as demonstrated in our case. Moreover, a recent study has shown how early infection control strategies may significantly reduce mortality associated with VRE bloodstream infections [18], underscoring the necessity of mandatory colonization prevention measures.

Another key point of discussion is the choice between standard culture-based screening and molecular tests. The former is undoubtedly widespread, cost-effective, and supported by large studies [24], which have highlighted that in low-incidence settings, with strict hygiene protocols, there is minimal difference in efficacy between molecular and culture-based screening.

However, a recent study following contacts of colonized patients showed that molecular testing allowed for a reduction in turnaround time by 5 to 27 days for positive rectal swabs, with a concordance rate of 97.5% between the two methods [27], and predictive positive and negative values of 100% and 98%, respectively. The results of this diagnostic assay have significant clinical implications, enabling prompt implementation of infection control measures. Rapid cohorting of carriers is crucial to prevent outbreaks and reduce the social impact and cost of infection control measures empirically implemented for all high-risk patients who ultimately test negative for MDRO colonization [28,29].

The limitations of this method are those typically associated with PCR techniques, including the high cost per sample compared to traditional methods, such as culture swabbing, and the potential for false positives due to the high sensitivity of the test. Due to its high sensitivity, false negatives are not expected. As PCR results do not provide susceptibility data, this test needs to be associated with standard cultures, which remains a critical component in guiding antibiotic therapy [27]. However, as demonstrated by our findings, this method offers a significantly reduced turnaround time (from 3 to 4 days to just 3 h), making it a valuable tool for preventing and reducing infection spread in high-intensity clinical settings with extensive nursing support.

There are limited data on the effectiveness of RT-PCR in this specific situation, and no studies demonstrate whether it may provide added value for infection control management in epidemic or high-prevalence epidemiological settings.

Study data on VRE colonization [11,15,18,23] are mainly based on rectal culture swabs for VRE detection. The use of PCR methods is mostly for research purposes rather than clinical practice, and indeed epidemiological data derive from cultural tests. To the best of our knowledge, this is the first report evaluating the clinical utility of PCR-based assay methods as a means to improve surveillance during an outbreak course.

### 4.3. Infection Control

The isolation of VRE-colonized patients from non-colonized individuals remains a cornerstone of infection control [29]. During outbreaks, dedicated units or cohorting strategies should be employed to minimize cross-contamination [25,30,31]. Moreover, contact precautions, including the use of gloves and gowns, must be rigorously implemented for all interactions with VRE-positive patients [32].

Ensuring compliance with hand hygiene protocols is equally critical [24]. Educational campaigns and regular audits can help maintain high adherence to these measures. Environmental contamination plays a significant role in VRE transmission. Routine and thorough cleaning of high-touch surfaces, combined with periodic terminal cleaning, is vital. Enhanced environmental decontamination protocols, such as double cleaning or the use of ultraviolet (UV) light and adenosine triphosphate detection systems, have proven effective in reducing contamination [33,34]. Some studies [31] have also demonstrated the impact of extensive renovations on reducing VRE colonization. For instance, a report [31] highlighted how the complete replacement of surfaces in a hematology unit led to a temporary but substantial decline in VRE transmission. However, recontamination of the environment eventually occurred, underscoring the importance of ongoing cleaning and monitoring efforts.

Due to the dynamic nature of outbreaks, continuous monitoring and adaptation of infection control strategies are crucial for their resolution. Surveillance data from the environment, combined with ongoing monitoring of patients, should be utilized to evaluate the effectiveness of interventions and identify areas requiring improvement, including environmental cleaning protocols, stricter cohorting methods with dedicated staff and segregated spaces, and hand hygiene reinforced through audits and staff training. In our experience, the coordinated management of the outbreak by all healthcare personnel, including nursing staff, medical professionals, infectious disease specialists, microbiologists, and the infection control team, was essential. We implemented all available preventive measures to identify colonized patients, reduce environmental contamination, and disrupt the chain of transmission.

The prompt resolution of the outbreak in our NICU demonstrates the efficacy of molecular testing as a quick surveillance tool that supports the appropriate application of infection control measures.

Our study provides a detailed analysis of strategies for controlling a VRE outbreak in the NICU, emphasizing the importance of rapid microbiological detection using molecular tests and the critical role of a dedicated hospital infection control team. While infection control measures and hygiene protocols are critical in preventing MDRO colonization, molecular tests may be valuable tools for improving surveillance and helping avoid pandemic outbreaks.

### 4.4. Limitations and Future Directions

This study has several limitations. Firstly, as a single-center, retrospective analysis, its findings may not be widely generalizable. Secondly, the small sample size limits our ability to draw definitive conclusions about the long-term outcomes of VRE-colonized neonates and to identify risk factors for developing VRE infections within a VRE-colonized population.

Future studies are needed to assess the effectiveness of systematic molecular screening for VRE in large and randomized cohorts in order to evaluate the cost–benefit factors of this approach. Based on our experience, systematic data are needed to update information, improve infection control systems within hospitals, and develop uniform guidelines based on epidemiological patterns. Finally, given the rising prevalence of multidrug-resistant organisms (MDROs), it is imperative to conduct epidemiological studies that provide clinicians with a better understanding of the true pathogenicity of VRE, thus guiding screening strategies while ensuring the efficient allocation of resources.

## 5. Conclusions

Although the pathogenicity of VRE is not fully understood, prevention strategies should prioritize minimizing colonization rates through rigorous hand hygiene, environmental cleaning, and effective antimicrobial stewardship programs. Furthermore, our data suggest that routine screening for VRE using molecular testing in high-risk or high-prevalence settings, such as NICUs, may be a valuable addition to infection control practices. This is particularly important during outbreaks or in endemic settings to reduce the risk of infection development and infection-related mortality.

This article is a revised and expanded version of a poster entitled “Vancomycin-resistant Enterococcus outbreak in neonatal intensive care unit: a real concern?” which will be presented in the poster session at 35th ESCMID Global 2025, Vienna, Austria, 11–15 April 2025 [35].

## Figures and Tables

**Table 1:** Baseline characteristics of the study population.

Patients, *n* (%)	21
Male, *n* (%)	13 (62)
Age at NICU admission (days), median (IQR)	6 (1–1)
Birthweight (g), median (IQR)	1879 (1150–2360)
Gestational age (weeks + days), median (IQR)	33 + 1 (31 + 3; 36 + 6)
Prematurity, *n* (%)	16 (76)
Prematurity (weeks + days), median (IQR)	31 + 3 (28 + 4; 34 + 2)
Reason for NICU admission, *n* (%)	
Prematurity	16 (76)
Congenital heart disease	7 (33)
Other	2 (10)
Time from admission to VRE colonization (days), median (IQR)	34 (6–37)
Previous BSI, *n* (%)	8 (38)
*Staphylococcus* spp.	7 (33)
*Streptococcus* spp.	1 (5)
*Enterobacterales* spp.	2 (10)
Previous antimicrobial treatment, *n* (%)	8 (38)
Vancomycin	7 (33)
Gentamycin	5 (24)
Meropenem	3 (14)
Linezolid	1 (5)
Orotracheal intubation, *n* (%)	6 (29)
Central venous catheter, *n* (%)	8 (38)
Surgical intervention, *n* (%)	7 (33)
Cardio-thorax	4 (19)
Abdominal	3 (14)
Oro-facial	1 (5)
Time from surgical intervention to colonization, *n* (%)	26 (16–36)
VRE infection, *n* (%)	1 (5)
Clinical response to infection treatment, *n* (%)	1 (100)
Molecular detection of *van A*, *n* (%)	8 (38)
Death, *n* (%)	1 (5)

**Table 2:** Antibiogram and molecular test.

Strains	Molecular Test	Antibiogram									
		Ampicillin		Tigecycline		Vancomycin		Teicoplanin		Linezolid	
		MIC (mg/L)	Interpretation	MIC (mg/L)	Interpretation	MIC (mg/L)	Interpretation	MIC (mg/L)	Interpretation	MIC (mg/L)	Interpretation
1	*van A*	>8	R	≤0.25	S	>256	R	96	R	2	S
2		>8	R	≤0.25	S	>256	R	48	R	≤1	S
3		>8	R	≤0.25	S	>256	R	96	R	≤1	S
4		>8	R	≤0.25	S	>256	R	>256	R	≤1	S
5		>8	R	≤0.25	S	>256	R	64	R	≤1	S
6		>8	R	≤0.25	S	>256	R	96	R	≤1	S
7		>8	R	≤0.25	S	>256	R	96	R	≤1	S
8		>8	R	≤0.25	S	>256	R	64	R	2	S
9		>8	R	≤0.25	S	>256	R	64	R	≤1	S
10		>8	R	≤0.25	S	>256	R	96	R	≤1	S
11		>8	R	≤0.25	S	>256	R	64	R	2	S
12		>8	R	≤0.25	S	>256	R	96	R	≤1	S
13	*van A*	>8	R	≤0.25	S	>256	R	96	R	≤1	S
14		>8	R	≤0.25	S	>256	R	>256	R	2	S
15	*van A*	>8	R	≤0.25	S	>256	R	64	R	2	S
16	*van A*	>8	R	≤0.25	S	>256	R	>256	R	2	S
17	*van A*	>8	R	≤0.25	S	>256	R	>256	R	2	S
18		>8	R	≤0.25	S	>32	R	>16	R	2	S
19	*van A*	>8	R	≤0.25	S	>32	R	>16	R	2	S
20	*van A*	>8	R	≤0.25	S	>32	R	>16	R	2	S
21	*van A*	>8	R	≤0.25	S	>32	R	>16	R	2	S

## Data Availability

The original contributions presented in the study are included in the article, further inquiries can be directed to the corresponding authors.
